# Self-Reported Work-Related Injury or Illness — Washington, 2011–2014

**DOI:** 10.15585/mmwr.mm6611a6

**Published:** 2017-03-24

**Authors:** Jennifer L. Marcum, Brian Chin, Naomi J. Anderson, David K. Bonauto

**Affiliations:** ^1^Safety and Health Assessment and Research for Prevention Program, Washington State Department of Labor and Industries; ^2^Department of Health Services, University of Washington, Seattle, Washington.

Work-related injuries and illnesses account for an estimated $250 billion annually in medical expenses and indirect costs, such as lost earnings and benefits, and reduced productivity at home; these costs are 12% more than the cost of all cancers and 30% more than costs for diabetes (*1*). Traditional state-wide surveillance systems often rely on employer-reported data to describe work-related injury and illness, which underestimate the magnitude. Studies estimate that the Bureau of Labor Statistics’ Survey of Occupational Injuries and Illnesses (BLS SOII) undercount 20%–70% of cases compared with workers’ compensation, which has also been shown to underestimate cases (*2*,*3*). These surveillance systems also lack information on potential individual-level risk factors, such as health status and risk behaviors. Data were analyzed from the Washington State Behavioral Risk Factor Surveillance System (WA BRFSS) to demonstrate an opportunity to enhance current occupational health surveillance systems. During 2011–2014, 6.4% of Washington workers reported work-related injuries or illnesses during the previous year. Work-related injuries or illnesses were significantly associated with industry and occupation, male gender, lower socioeconomic status, chronic health conditions, and substance use. Because BRFSS does not rely on employer report and contains information on workers not available in traditional occupational health surveillance systems, it is a useful tool for identifying and examining work-related injury and illness.

BRFSS is a CDC-sponsored, statewide telephone survey conducted annually to collect information on health outcomes and behaviors. The sample includes adults aged ≥18 years in a private residence or college housing. Since 1995, the WA BRFSS has added questions* to collect information on industry and occupation. Trained coders assign industry and occupation codes to verbatim responses through automated and manual coding processes. During 2011–2014, WA BRFSS also collected work-related injury or illness information on working adults with a state-added question.^†^ The response rates in Washington during this period ranged from 31% to 44%.

Among the 51,335 respondents to the 2011–2014 WA BRFSS, 25,493 (50.0%) were eligible to answer the work-related injury or illness question, including those currently employed for wages (20,028, 78.5%), self-employed (4,059, 15.9%), and out of work for <1 year (1,406, 5.5%). Among all eligible respondents, 24,650 (96.7%) participated in the optional work-related injury or illness module.

Associations between work-related injury or illness and select demographics, health conditions,^§^ and risk behaviors^¶^ were examined. Results were weighted to the adult population in Washington. Statistical significance was determined using Rao-Scott chi-square tests, at α=0.05.

During 2011–2014, an estimated 6.4% (190,076 annually) of employed Washington residents reported having a work-related injury or illness during the previous year (Table 1). The percentage of workers with work-related injuries or illnesses varied significantly by respondent’s reported industry and occupation, with the highest prevalences reported among workers in the Transportation and Warehousing (9.2%), and Construction industries (8.9%), and the Installation, Repair, and Maintenance (11.1%), Service (9.7%), and Transportation and Material Moving (9.6%) occupations (Table 1). The percentage of workers reporting work-related injury or illness was lowest among females (5.7%), married persons (5.4%), persons with ≥4 years of college (4.1%); and persons with an annual household income ≥$75,000 (4.4%) (Table 2).

**TABLE 1:** Self-reported work-related injury or illness among employed adults, by year and employment characteristics — WA BRFSS, Washington, 2011–2014

Characteristic	No. in sample	Weighted percent with work- injury/illness	(95% CI)	p value
**Total**	24,650	6.4	(5.9–6.8)	—
**Year**				
2011	6,884	6.0	(5.1–6.9)	0.568
2012	7,643	6.4	(5.2–7.2)	—
2013	5,367	6.9	(6.0–7.8)	—
2014	4,756	6.2	(5.2–7.2)	—
**Employment status**				
Employed for wages	19,345	6.5	(6.1–7.0)	0.013
Self employed	3,975	4.7	(3.6–5.7)	—
Out of work for <1 yr	1,330	7.5	(5.4–9.5)	—
**Industry** *				
Agriculture, forestry, fishing, hunting, and mining	968	7.8	(5.3–10.4)	<0.001
Construction	1,361	8.9	(6.8–11.0)	—
Manufacturing	2,255	7.2	(5.7–8.6)	—
Wholesale and retail trade	2,236	6.2	(4.8–7.6)	—
Transportation and warehousing	915	9.2	(6.7–11.7)	—
Utilities	269	7.1	(3.3–10.9)	—
Information, finance and insurance, real estate services, and management	1,945	3.0	(1.7–3.8)	—
Professional, scientific, and technical services	2,217	2.9	(1.8–3.9)	—
Administrative support and waste management services	659	7.7	(4.5–10.8)	—
Educational services	2,776	5.4	(4.2–6.6)	—
Health care and social assistance	3,699	6.3	(5.2–7.4)	—
Arts, entertainment, and recreation	496	6.8	(3.7–10.0)	—
Accommodation and food services	718	7.6	(4.9–10.3)	—
Other services	1,112	6.1	(4.0–8.2)	—
Public administration	1,744	7.4	(5.8–9.0)	—
**Occupation** **^†^**				
Management, business and financial	4,759	3.7	(2.9–4.5)	<0.001
Professional and related	7,375	4.5	(3.8–5.2)	—
Service	2,968	9.7	(8.1–11.2)	—
Sales and related	1,704	3.9	(2.6–5.1)	—
Office and administrative support	2,396	5.1	(3.8–6.4)	—
Farming, fishing, and forestry	369	7.8	(4.2–11.4)	—
Construction and extraction	849	9.4	(6.8–11.9)	—
Installation, repair, and maintenance	577	11.1	(8.1–14.2)	—
Production	965	7.9	(6.0–9.9)	—
Transportation and material moving	1,070	9.6	(7.2–12.0)	—

**Abbreviations:** CI = confidence interval**;** WA BRFSS = Washington State Behavioral Risk Factor Surveillance System.

* North American Industrial Classification System, Industry Sectors.

^†^ Standard Occupational Classifications.

**TABLE 2:** Self-reported work-related injury or illness among employed adults, by demographic characteristics — WA BRFSS, Washington, 2011–2014

Characteristic	No. in sample	Weighted percent with work- injury/illness	(95% CI)		p value	
**Total**	**24,650**	**6.4**	**(5.9–6.8)**		—	
**Sex**						
Male	11,715	6.9		(6.3–7.6)	0.005	
Female	12,935	5.7		(5.1–6.3)	—	
**Age group (yrs)**						
18–24	1,286	6.2		(4.6–7.7)	0.045	
25–34	2,906	7.0		(5.8–8.1)	—	
35–44	4,474	5.7		(4.8–6.6)	—	
45–54	6,356	6.7		(5.9–7.5)	—	
55–64	6,958	6.7		(5.9–7.6)	—	
≥65	2,408	3.7		(2.6–4.8)	—	
**Race/Ethnicity** *						
White	20,401	6.3		(5.8–6.7)		0.111
Black or African American	405	8.8		(5.6–12.0)		—
Asian	545	4.6		(2.4–6.8)		—
Native Hawaiian/Other Pacific Islanders	408	5.4		(2.5–8.3)		—
American Indian/Alaskan Native	181	9.5		(2.6–12.8)		—
Other	247	9.3		(3.4–15.6)		—
Multiracial	490	9.6		(5.7–13.5)		—
Hispanic	1,684	6.5		(5.0–8.0)		—
**Marital status**						
Married	14,877	5.4		(4.9–5.8)	<0.001	
Divorced	3,481	9.6		(8.0–11.2)	—	
Widowed	1,355	8.7		(6.1–11.3)	—	
Separated	3,741	6.4		(5.3–7.5)	—	
Never married	1,052	7.4		(5.4–9.5)	—	
**Children**						
Yes	15,819	6.5		(5.9–7.1)	0.480	
No	8,759	6.2		(5.5–6.8)	—	
**Education**						
< High school diploma	1,067	8.1		(5.9–10.3)	<0.001	
High school graduate	4,706	7.2		(6.2–8.2)	—	
College 1–3 yrs	7,192	7.5		(6.7–8.3)	—	
College ≥4 yrs	11,648	4.1		(3.6–4.5)	—	
**Income** (dollars)						
<20,000	1,745	7.2		(5.5–8.9)	<0.001	
20,000–<25,000	1,403	7.3		(5.4–9.2)	—	
25,000–<35,000	1,994	8.5		(6.7–10.4)	—	
35,000–<50,000	3,140	8.5		(7.0–9.9)	—	
50,000–<75,000	4,399	6.7		(5.7–7.7)	—	
≥75,000	9,829	4.4		(3.8–5.0)	—	
**Veteran status (ever)**						
Yes	2,717	9.2		(7.7–10.7)	<0.001	
No	21,913	6.0		(5.6–6.5)	—	
**Sexual orientation**						
Heterosexual	22,976	6.4		(5.9–6.8)	0.719	
Homosexual, bisexual, or other	894	6.0		(4.0–8.8)	—	

**Abbreviations:** CI = confidence interval**;** WA BRFSS = Washington State Behavioral Risk Factor Surveillance System.

* Race/ethnicity was coded into mutually exclusive categories.

The percentage of respondents reporting work-related injuries or illnesses was higher among persons with chronic health conditions, such as heart disease, depression, arthritis, blindness or difficulty seeing, and asthma, than among workers not reporting these conditions (Table 3). Reporting of these health conditions was not significantly higher among workers within high-risk industries and occupations (≥7 work-related injuries or illnesses reported per 100 workers) compared with workers in lower-risk (<7 per 100 workers) industries and occupations (data not shown).

**TABLE 3:** Self-reported work-related injury or illness among employed adults, by selected health and behavior characteristics — WA BRFSS, Washington, 2011–2014

**Characteristic**	**No. in sample**	**Weighted percent with work- injury/illness**	**(95% CI)**	**p value**
**Total**	**24,105**	**6.3**	**(5.9–6.8)**	—
**Body mass index (BMI)**				
Underweight and normal (BMI<25.0)	8,344	5.8	(5.0–6.5)	0.045
Overweight (25.0≤BMI<30.0)	8,611	6.5	(5.8–7.3)	—
Obese (BMI ≥30.0)	6,370	7.3	(6.4–8.1)	—
**Coronary heart disease (ever)**				
Yes	512	9.5	(5.9–13.2)	0.038
No	24,048	6.3	(5.9–6.8)	—
**Diabetes (ever)**				
Yes	1,937	7.6	(5.8–9.4)	0.125
No	22,684	6.3	(5.8–6.7)	—
**Depression (ever)**				
Yes	4,710	10.1	(8.8–11.4)	<0.001
No	19,843	5.5	(5.0–5.9)	—
**Arthritis (ever)**				
Yes	5,554	10.4	(9.1–11.6)	<0.001
No	18,959	5.5	(5.1–6.0)	—
**Blind or serious difficulty seeing**				
Yes	2,649	10.6	(8.8–12.4)	<0.001
No	21,757	5.9	(5.4–6.3)	—
**Asthma (current)**				
Yes	2,098	8.2	(6.7–9.8)	0.007
No	22,364	6.2	(5.7–6.7)	—
**Average hours of sleep in 24-hour period (2013–2014 only)**				
≤6	3,090	9.4	(8.0–10.9)	<0.001
>6	6,975	5.0	(4.4–5.7)	—
**Use pain killer to get high (any use in past 30 days)**				
Yes	135	15.9	(6.5–25.2)	0.002
No	21,131	6.2	(5.8–6.7)	—
**Marijuana use (any use in past 30 days)**				
Yes	1,428	8.9	(6.9–10.8)	0.002
No	19,758	6.1	(5.6–6.6)	—
**Smoker (current)**				
Yes	3,168	10.0	(8.6–11.4)	<0.001
No	21,191	5.6	(5.2–6.1)	—
**Binge drinking (male: ≥5drinks; female: ≥4 drinks, on any occasion)**				
Yes	4,169	7.4	(6.3–8.4)	0.023
No	19,852	6.1	(5.6–6.6)	—

**Abbreviations:** CI = confidence interval**;** WA BRFSS = Washington State Behavioral Risk Factor Surveillance System.

The percentage of work-related injury or illness was significantly higher among workers who reported sleeping ≤6 hours per night on average (9.4%) compared with workers who slept an average of >6 hours per night (5.0%) (Table 3). The percentage of workers who reported work-related injury or illness was significantly higher among respondents who reported use of pain killers to get high (15.9%) or marijuana (8.9%), being a current smoker (10.0%), and binge drinking (7.4%), compared with workers who did not report these behaviors (Table 3).

## Discussion

This is the first study to demonstrate the utility of the BRFSS as an occupational health surveillance system by examining associations of work-related injuries or illnesses with selected worker demographics, health conditions, and behaviors. The associations reported here are corroborated elsewhere in the literature (*4*–*7*), further supporting the use of BRFSS as a potential surveillance tool. For example, the industries with the highest percentages of work-related injury or illness identified in this report are consistent with high-risk industries reported from other data sources, including Transportation and Warehousing; Construction; and Agriculture, Forestry, Fishing, Hunting, and Mining (*4*). Work injury and illness disparities by gender, education, and income described here replicate a body of evidence demonstrating higher unintentional injury risk among males and the relation of lower income and education attainment with overall poor health status (*5*). The WA BRFSS data presented in this analysis also reproduced important associations between several chronic conditions, such as obesity, heart disease, depression, arthritis, asthma, and poor eyesight and work-related injury and illness that have been documented by other studies (*6*,*7*).

Higher percentages of work-injury and illness among persons reporting an average of ≤6 hours of sleep per night, binge drinking, and recent use of painkillers to get high and marijuana compared with persons not reporting those conditions have also been identified as risk factors for work-related injury or illness in other studies (*7*,*8*). Marijuana and pain killer usage was measured by reported behavior in the previous month only, whereas work-related injury and illness was measured over an entire year. This suggests that substance use might also be an outcome of work-related injuries and illnesses rather than solely a risk factor, because opioids are frequently prescribed to treat injured workers (*9*).

The findings in this report are subject to at least five limitations. First, BRFSS findings are limited because of the survey’s cross-sectional design. This prevents identification of causal factors for work-related injury or illness and the ability to determine whether reported health conditions existed before, or resulted from a work-related injury or illness. Second, because responses are self-reported, the findings are also subject to recall and social desirability biases, which could result in differential recall of more severe or recent events. Third, the survey question used to collect reports of work-related injury or illness here prevents characterization by severity or distinguishing conditions. Fourth, workers' or physicians' definitions of a work-related injury or illness might differ from legally reportable definitions, so results are not directly comparable to state-level employer-reported data, such as the BLS SOII. Finally, BRFSS does not collect information on other factors known to cause work-related injuries and illnesses such as physical, chemical, biological or ergonomic hazards.

This report demonstrates the utility of the WA BRFSS as a statewide occupational health surveillance system, which unlike other current surveillance systems, collects work-related injury or illness data. The WA BRFSS identifies cases by worker-report, and therefore is not subject to the same underreporting biases present in systems that rely on physician or employer reports of injury and illness. The WA BRFSS also collects demographic, health status and behavior information on workers that is not available in other sources of occupational injury and illness data, allowing for more complete characterization of persons with recent work-related injuries and illnesses. The WA BRFSS could serve as a model for other states to include similar questions to collect work-related injury and illness data to enhance their occupational surveillance capabilities, and allow for opportunities to aggregate state data for evaluation of this outcome on a larger scale. Further research might help to determine if there is segregation of workers by their demographic, health, and behavior characteristics into high-risk industries and occupations, or if these characteristics are causally related to injury and illness. Assessment of health status and behaviors as potential contributors to occupational injury risk might inform future prevention activities, but does not mitigate the employer’s responsibility in providing a workplace free from hazards.

**Summary**

**What is already known about this topic?**

Work-related injuries and illnesses are frequent and have lasting negative economic and social consequences. Comprehensive surveillance is critical for identifying and evaluating effective control strategies and populations at risk.

**What is added by this report?**

Data from the Washington State Behavioral Risk Factor Surveillance System (WA BRFSS) were used to gather information on work-related injury or illness. During 2011–2014, 6.4% of Washington workers reported work-related injuries or illnesses during the previous year. Work-related injuries or illnesses were significantly associated with industry and occupation, male gender, lower socioeconomic status, chronic health conditions, and substance use.

**What are the implications for public health practice?**

Because BRFSS does not rely on employer report and contains information on workers not available in traditional occupational health surveillance systems, it is a useful tool for identifying and examining work-related injury and illness. BRFSS provides opportunities to enhance ability to track injury and illness trends, identify and describe disparities among workers by industry and occupation of employment, and generate hypotheses for control measures. Future research should consider further assessment of health status as a potential contributor to occupational injury risk.

## Footnotes

* “What is your job title?” and “What kind of work do you do?”

† Work-related injury or illness defined as a “yes” response to “In the past 12 months, have you been injured while performing your job, or has a doctor or other medical professional told you that you have a work-related illness?”

§ Coronary heart disease defined as a “yes” response to “Has a doctor, nurse, or other health professional ever told you that you had angina or coronary heart disease?”; Diabetes defined as a “yes” response to “Has a doctor, nurse, or other health professional ever told you that you have diabetes?”; Depression defined as a “yes” response to “Has a doctor, nurse, or other health professional ever told you that you have a depressive disorder, including depression, major depression, dysthymia, or minor depression?”; Arthritis defined as a “yes” response to “Has a doctor, nurse, or other health professional ever told you that you have some form of arthritis, rheumatoid arthritis, gout, lupus, or fibromyalgia?”; Blindness or serious difficulty seeing defined as a “yes” response to “Are you blind, or do you have serious difficulty seeing, even when wearing glasses?”; Asthma defined as a “yes” response to “Has a doctor, nurse, or other health professional ever told you that you had asthma?”, and a “yes” response to “Do you still have asthma?”

¶ Average hours of sleep in 24-hour period defined as the response to “On average, how many hours of sleep do you get in a 24-hour period?”; Use of pain killer to get high defined as a response >0 to “During the past 30 days, on how many days did you use a pain killer to get high, like Vicodin, OxyContin (sometimes called Oxy or OC) or Percocet (sometimes called Percs)?”; Marijuana usage defined as a response >0 to “During the past 30 days, on how many days did you use marijuana or hashish (grass, hash, or pot)?”; Smoker defined as a “yes” response to “Have you smoked at least 100 cigarettes in your entire life?”, and an “every day” or “some days” response to “Do you now smoke cigarettes every day, some days, or not at all?”; Binge drinking defined as a response >0 to “During the past 30 days, how many days per week or per month did you have at least one drink of any alcoholic beverage such as beer, wine, a malt beverage or liquor?”, and a response >0 to “Considering all types of alcoholic beverages, how many times during the past 30 days did you have [5 or more drinks for men/4 or more drinks for women] on an occasion?”
